# Supplementary material for: Phylogenetic and recombination analyses of two deformed wing virus strains from different honeybee species in China
Source: PeerJ. 2019 Jun 28;7:e7214. doi: 10.7717/peerj.7214 (PMC6601602; doi:10.7717/peerj.7214)
Supplement: Supplemental Information 1 — The values were calculated using DNASTAR. Values above and below the diagonal frames indicate the percentage of amino acid identity and nucleotide sequence identity, respectively. [file peerj-07-7214-s001.docx]

|  | **D1** | **D2** | **D3** | **D4** | **D5** | **D6** | **D7** | **D8** | **D9** | **D10** | **D11** | **D12** | **D13** | **D14** | **D15** | **D16** | **D17** | **D18** | **D19** | **D20** | **D21** | **D22** |
| --- | --- | --- | --- | --- | --- | --- | --- | --- | --- | --- | --- | --- | --- | --- | --- | --- | --- | --- | --- | --- | --- | --- |
| **D1** |  | **(99.4)** | **(98.7)** | **(98.9)** | **(99.1)** | **(99.0)** | **(99.0)** | **(99.1)** | **(98.0)** | **(98.7)** | **(98.2)** | **(97.6)** | **(95.2)** | **(95.3)** | **(97.9)** | **(97.3)** | **(97.3)** | **(98.1)** | **(97.3)** | **(97.3)** | **(97.8)** | **(97.5)** |
| **D2** | **98.5** |  | **(99.0)** | **(99.1)** | **(99.4)** | **(99.1)** | **(99.3)** | **(99.2)** | **(98.2)** | **(98.9)** | **(98.4)** | **(97.8)** | **(95.4)** | **(95.6)** | **(98.2)** | **(97.5)** | **(97.5)** | **(98.4)** | **(97.4)** | **(97.4)** | **(97.9)** | **(97.7)** |
| **D3** | **97.7** | **98.8** |  | **(98.5)** | **(98.6)** | **(98.6)** | **(98.7)** | **(98.4)** | **(97.5)** | **(98.1)** | **(97.9)** | **(97.2)** | **(94.7)** | **(94.9)** | **(97.5)** | **(96.9)** | **(96.8)** | **(97.6)** | **(96.8)** | **(96.8)** | **(97.3)** | **(97.1)** |
| **D4** | **97.9** | **98.0** | **97.3** |  | **(99.0)** | **(98.8)** | **(98.9)** | **(98.8)** | **(98.0)** | **(98.4)** | **(98.2)** | **(97.5)** | **(95.1)** | **(95.3)** | **(98.0)** | **(97.2)** | **(97.2)** | **(98.0)** | **(97.2)** | **(97.2)** | **(97.8)** | **(97.3)** |
| **D5** | **97.7** | **97.8** | **96.9** | **97.3** |  | **(99.0)** | **(99.1)** | **(99.1)** | **(98.3)** | **(98.8)** | **(98.4)** | **(97.8)** | **(95.4)** | **(95.5)** | **(98.1)** | **(97.3)** | **(97.3)** | **(98.4)** | **(97.4)** | **(97.4)** | **(98.0)** | **(97.7)** |
| **D6** | **97.9** | **98.5** | **97.4** | **97.6** | **97.6** |  | **(99.0)** | **(98.7)** | **(98.2)** | **(98.4)** | **(98.4)** | **(97.7)** | **(95.3)** | **(95.4)** | **(97.9)** | **(97.3)** | **(97.2)** | **(98.1)** | **(97.2)** | **(97.1)** | **(98.0)** | **(97.8)** |
| **D7** | **98.0** | **98.5** | **97.7** | **97.5** | **97.4** | **97.9** |  | **(99.0)** | **(98.1)** | **(98.5)** | **(98.2)** | **(97.6)** | **(95.2)** | **(95.4)** | **(98.1)** | **(97.2)** | **(97.2)** | **(98.1)** | **(97.2)** | **(97.2)** | **(97.8)** | **(97.5)** |
| **D8** | **97.8** | **98.3** | **97.4** | **97.3** | **97.1** | **97.8** | **97.6** |  | **(97.9)** | **(98.7)** | **(98.1)** | **(97.5)** | **(95.3)** | **(95.4)** | **(98.0)** | **(97.4)** | **(97.3)** | **(98.0)** | **(97.3)** | **(97.3)** | **(97.7)** | **(97.5)** |
| **D9** | **96.0** | **96.3** | **95.6** | **95.8** | **96.3** | **96.1** | **95.9** | **96.1** |  | **(98.0)** | **(98.1)** | **(97.7)** | **(95.3)** | **(95.4)** | **(97.5)** | **(96.8)** | **(96.8)** | **(97.6)** | **(96.8)** | **(96.7)** | **(98.6)** | **(98.4)** |
| **D10** | **96.8** | **97.0** | **96.4** | **96.3** | **97.1** | **96.5** | **96.6** | **96.8** | **96.6** |  | **(98.2)** | **(97.5)** | **(95.0)** | **(95.2)** | **(97.8)** | **(97.0)** | **(97.0)** | **(97.9)** | **(96.9)** | **(96.9)** | **(97.7)** | **(97.5)** |
| **D11** | **96.4** | **96.7** | **96.0** | **96.1** | **96.5** | **96.7** | **96.3** | **96.4** | **96.2** | **96.6** |  | **(97.6)** | **(95.0)** | **(95.1)** | **(97.6)** | **(96.8)** | **(96.8)** | **(97.6)** | **(96.8)** | **(96.8)** | **(97.7)** | **(97.5)** |
| **D12** | **95.9** | **96.3** | **95.6** | **95.6** | **96.2** | **96.1** | **95.9** | **95.9** | **96.0** | **96.2** | **96.6** |  | **(94.4)** | **(94.5)** | **(97.0)** | **(96.2)** | **(96.2)** | **(97.0)** | **(96.1)** | **(96.1)** | **(97.3)** | **(97.1)** |
| **D13** | **84.2** | **84.5** | **84.2** | **84.3** | **84.6** | **84.2** | **84.5** | **84.4** | **84.2** | **84.3** | **83.9** | **83.8** |  | **(99.7)** | **(96.4)** | **(97.5)** | **(97.5)** | **(96.7)** | **(97.5)** | **(97.4)** | **(94.8)** | **(94.7)** |
| **D14** | **84.3** | **84.6** | **84.4** | **84.4** | **84.7** | **84.4** | **84.7** | **84.6** | **84.2** | **84.5** | **83.9** | **83.9** | **99.3** |  | **(96.4)** | **(97.5)** | **(97.5)** | **(96.9)** | **(97.5)** | **(97.5)** | **(94.9)** | **(94.9)** |
| **D15** | **91.6** | **92.0** | **91.4** | **91.4** | **91.1** | **91.3** | **91.7** | **91.5** | **90.3** | **90.5** | **90.3** | **89.9** | **91.1** | **91.0** |  | **(98.5)** | **(98.5)** | **(99.2)** | **(98.4)** | **(98.4)** | **(97.2)** | **(97.0)** |
| **D16** | **90.3** | **90.5** | **89.9** | **90.0** | **90.7** | **90.2** | **90.4** | **90.4** | **89.9** | **90.0** | **89.7** | **89.4** | **92.4** | **92.2** | **96.0** |  | **(100.0)** | **(98.7)** | **(99.5)** | **(99.5)** | **(96.5)** | **(96.4)** |
| **D17** | **90.3** | **90.4** | **89.9** | **90.0** | **90.6** | **90.2** | **90.3** | **90.2** | **89.9** | **90.0** | **89.7** | **89.4** | **92.4** | **92.1** | **96.0** | **100.0** |  | **(98.6)** | **(99.5)** | **(99.4)** | **(96.5)** | **(96.3)** |
| **D18** | **93.1** | **93.3** | **92.6** | **92.9** | **93.7** | **93.1** | **93.0** | **92.7** | **92.1** | **92.4** | **92.1** | **91.9** | **89.4** | **89.4** | **95.8** | **95.2** | **95.1** |  | **(98.9)** | **(98.8)** | **(97.3)** | **(97.1)** |
| **D19** | **90.7** | **91.1** | **90.5** | **90.6** | **91.8** | **90.7** | **90.9** | **90.8** | **90.4** | **90.8** | **90.4** | **90.2** | **91.6** | **91.3** | **94.8** | **96.4** | **96.4** | **96.9** |  | **(100.0)** | **(96.4)** | **(96.3)** |
| **D20** | **90.7** | **90.9** | **90.4** | **90.6** | **91.6** | **90.7** | **90.6** | **90.5** | **90.4** | **90.7** | **90.4** | **90.1** | **91.5** | **91.3** | **94.7** | **96.4** | **96.4** | **96.8** | **99.9** |  | **(96.4)** | **(96.3)** |
| **D21** | **96.0** | **96.4** | **95.7** | **95.7** | **96.4** | **96.1** | **96.0** | **96.0** | **96.6** | **96.3** | **95.9** | **95.6** | **84.3** | **84.3** | **90.3** | **90.2** | **90.1** | **92.2** | **90.6** | **90.4** |  | **(98.1)** |
| **D22** | **95.9** | **96.2** | **95.5** | **95.5** | **96.2** | **96.0** | **95.9** | **96.0** | **96.5** | **96.3** | **96.2** | **95.8** | **84.3** | **84.3** | **90.1** | **89.9** | **89.9** | **92.2** | **90.4** | **90.4** | **96.7** |  |
